# Sorting permutations by cut-circularize-linearize-and-paste operations

**DOI:** 10.1186/1471-2164-12-S3-S26

**Published:** 2011-11-30

**Authors:** Keng-Hsuan Huang, Kun-Tze Chen, Chin Lung Lu

**Affiliations:** 1 Institute of Bioinformatics and Systems Biology, National Chiao Tung University, Hsinchu 30010, Taiwan; 2 Department of Computer Science, National Tsing Hua University, Hsinchu 30013, Taiwan

## Abstract

**Background:**

Genome rearrangements are studied on the basis of genome-wide analysis of gene orders and important in the evolution of species. In the last two decades, a variety of rearrangement operations, such as reversals, transpositions, block-interchanges, translocations, fusions and fissions, have been proposed to evaluate the differences between gene orders in two or more genomes. Usually, the computational studies of genome rearrangements are formulated as problems of sorting permutations by rearrangement operations.

**Result:**

In this article, we study a sorting problem by cut-circularize-linearize-and-paste (CCLP) operations, which aims to find a minimum number of CCLP operations to sort a signed permutation representing a chromosome. The CCLP is a genome rearrangement operation that cuts a segment out of a chromosome, circularizes the segment into a temporary circle, linearizes the temporary circle as a linear segment, and possibly inverts the linearized segment and pastes it into the remaining chromosome. The CCLP operation can model many well-known rearrangements, such as reversals, transpositions and block-interchanges, and others not reported in the biological literature. In addition, it really occurs in the immune response of higher animals. To distinguish those CCLP operations from the reversal, we call them as non-reversal CCLP operations. In this study, we use permutation groups in algebra to design an *O*(*δn*) time algorithm for solving the weighted sorting problem by CCLP operations when the weight ratio between reversals and non-reversal CCLP operations is 1:2, where *n* is the number of genes in the given chromosome and *δ* is the number of needed CCLP operations.

**Conclusion:**

The algorithm we propose in this study is very simple so that it can be easily implemented with 1-dimensional arrays and useful in the studies of phylogenetic tree reconstruction and human immune response to tumors.

## Background

Genome rearrangements are studied on the basis of genome-wide analysis of gene orders and important in the evolution of species [1-6]. Since a DNA molecule has two strands, a gene in the genome rearrangement studies is usually denoted by a signed integer, with sign indicating the DNA strand to which the gene belongs, and a chromosome by a series of integers corresponding to those genes on the chromosome. In the last two decades, a variety of rearrangement operations have been proposed to evaluate the differences between gene orders in two or more genomes. Basically, these operations can be classified into two categories: (1) ‘intra-chromosomal’ rearrangements, such as reversals, transpositions and block-interchanges (also called ‘generalized transpositions’), and (2) ‘inter-chromosomal’ rearrangements, such as fusions, fissions and translocations. *Reversals*, often called *inversions* in the biological literature, rearrange a segment of continuous integers on the chromosome by reversing the order of the integers and changing their signs [3,7-11]. *Transpositions* act on two adjacent and non-overlapping segments on the chromosome by exchanging their locations [10,12-15]. *Block-interchanges* function as a *generalized transposition* that exchanges two non-overlapping but not necessarily adjacent segments on the chromosome [11,15-19]. *Translocations* affect two chromosomes by exchanging their end segments [2,11,20-22]. *Fusions* merge two chromosomes into one chromosome and *fissions* split a chromosome into two chromosomes [2,11,13,18].

**Figure 1:**
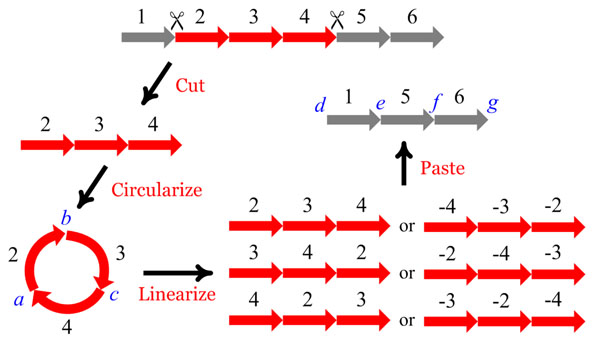
**Illustration of a cut-circularize-linearize-and-paste operation.** A modified cut-circularize-linearize-and-paste operation that can model seven different kinds of rearrangement, where the cutting site of the temporary circle with genes 2, 3 and 4 can be either *a*, *b* or *c*, and the inserting place of the linearized segment at the remaining chromosome can be either *d*, *e*, *f* or *g*.

Recently, great attention has been paid to the study of genome rearrangement using block-interchanges, since block-interchanges contain transpositions as a special case and, currently, the computational models involving block-interchanges are more tractable than those involving transpositions. More recently, Yancopoulos et al. defined a double cut and join (DCJ) operation that can model all the rearrangement operations described previously [23]. The DCJ is an operation that cuts one or two chromosomes in two places and rejoins the four broken ends in a new way. Intriguingly, block-interchanges, as well as transpositions, can be modeled by two consecutive DCJ operations, while others by one DCJ operation. In fact, as mentioned in [24], the two consecutive DCJ operations can be viewed as the following procedure to model transpositions or block-interchanges. (1) Excision: cut a segment from a chromosome that can be linear or circular. (2) Circularization: join the ends of the excised segment into a temporary circle. (3) Linearization: cut the temporary circle in any place as a linear segment. (4) Reincorporation: paste the linearized segment back to the remaining chromosome at a new site. As also pointed out in [24], this process of fragment excision, circularization, linearization and reincorporation indeed occurs in the immune response of higher animals. Here, we make a little modification to the reincorporation step in the above process by allowing the linearized segment to be possibly inverted before its reinsertion and also allowing inverted or non-inverted linearized segment to be pasted back to the remaining chromosome at any site (see Figure 1 for the modified model). This modification enables the above cut-circularize-linearize-and-paste (CCLP for short) operation to model seven different kinds of rearrangements, as will be detailed below. It is interesting to note that in addition to transposition and block-interchange, a CCLP operation can model reversal, *inverted transposition* (or *transversal*) [10] and others that are currently not reported in the biological literature. The seven rearrangements modeled by the CCLP operation are described as follows (see Figure 1 for a reference).

• Case I – reversal:

As illustrated in Figure 1, a segment with genes 2, 3 and 4 is cut from a chromosome (1,2,3,4,5,6) and joined as a temporary circle, which is then cut in the same place as it was created by the join (i.e., the *a* site in Figure 1), and inverted and pasted back to the chromosome at the cutting site (i.e., the *e* site in Figure 1). As a result, this CCLP operation performs as a reversal that changes the chromosome (1,2,3,4,5,6) into (1,-4,-3,-2,5,6).

• Case II – transposition:

The temporary circle is cut in a new place (e.g., the *b* site in Figure 1) and pasted back to the chromosome at the cutting site. This CCLP operation performs as a transposition that changes (1,2,3,4,5,6) into (1,3,4,2,5,6).

• Case III – two consecutive, adjacent reversals:

The temporary circle is cut in a new place (e.g., the *b* site in Figure 1), and then inverted and pasted back to the chromosome at the cutting site. This CCLP operation changes (1,2,3,4,5,6) into (1,-2,-4,-3,5,6), which is equivalent to that (1,2,3,4,5,6) is first changed into (1,2,-4,-3,5,6) by a reversal, which is further changed into (1,-2,-4,-3,5,6) by another reversal. Note that the chromosomal regions affected by these two consecutive reversals are adjacent.

• Case IV – transposition:

The temporary circle is cut in the same place as it was joined and then pasted back to the chromosome at a new site (e.g., the *f* site in Figure 1). This CCLP operation performs as a transposition that changes (1,2,3,4,5,6) into (1,5,2,3,4,6).

• Case V – transversal:

The temporary circle is cut in the same place as it was joined, and then inverted and pasted back to the chromosome at a new site (e.g., the *f* site in Figure 1). This CCLP operation performs as an inverted transposition (i.e., transversal) that changes (1,2,3,4,5,6) into (1,5,-4,-3,-2,6).

• Case VI – block-interchange:

The temporary circle is cut in a new place (e.g., the *b* site in Figure 1) and then pasted back to the chromosome at a new site (e.g., the *f* site in Figure 1). This CCLP operation performs as a block-interchange that changes (1,2,3,4,5,6) into (1,5,3,4,2,6).

• Case VII – two consecutive, overlapping reversals:

The temporary circle is cut in a new place (e.g., the *b* site in Figure 1), and then inverted and pasted back to the chromosome at a new site (e.g., the *f* site in Figure 1). This CCLP operation changes (1,2,3,4,5,6) into (1,5,-2,-4,-3,6), which is equivalent to that (1,2,3,4,5,6) is first changed into (1,2,-5,-4,-3,6) by a reversal, which is further changed into (1,5,-2,-4,-3,6) by another reversal. Note that the chromosomal regions affected by these two consecutive reversals are overlapping.

All these seven rearrangements described above are simply called *CCLP operations.* But, to distinguish those CCLP operations from the reversal, we call them as *non-reversal CCLP operations* in the sequel of this paper. In this article, we are interested in designing efficient algorithms to solve the genome rearrangement problem involving all the seven CCLP operations. If all these CCLP operations are weighted equally, the problem aims to find a minimum number of operations to sort a signed permutation of representing a chromosome. In this case, however, non-reversal CCLP operations are favored in the rearrangement scenario of the optimal solution, as will be clear later, which contradicts with the observation made by biologists that in most organisms, reversals are observed much more frequently when compared with other rearrangements. Therefore, it may require a reversal to be weighted differently from other CCLP operations. In this circumstance, the problem is then called *weighted sorting problem by CCLP operations*, which is to find a series of CCLP operations whose weight sum is minimum. In this study, we pay our attention on the case in which the weight ratio between reversals and non-reversal CCLP operations is 1:2 and use the permutation group in algebra to design an *O*(*δn*) time algorithm for solving the problem, where *n* is the number of genes in the given chromosome and *δ* is the number of needed CCLP operations.

## Preliminaries

Below, we introduce some definitions about the basics of permutation groups, as well as a couple of lemmas from Huang and Lu [11], that are useful for the study of genome rearrangements. Let *E* = {1, 2, …, *n*} be a set of *n* positive integers. Then a permutation of *E* is defined as a one-to-one function from *E* into itself and can simply be denoted by a product of some cycles. For example, a permutation expressed as *α* = (1, 6, 4) (2, 5, 3) means that *α*(1) = 6, *α*(6) = 4, *α*(4) = 1, *α*(2) = 5, *α*(5) = 3 and *α*(3) = 2. Basically, a cycle is cyclic and hence it does not matter which element in the cycle is written as the first. If the cycles in a permutation are all *disjoint* (i.e., any two cycles have no common elements), then their product is called the *cycle decomposition.* If a cycle has *k* elements, then it is called a *k-cycle.* The element in a 1-cycle is usually called *fixed.* It is a convention that the 1-cycles in a permutation are not written explicitly. If all the elements in *E* are fixed in a permutation, then this permutation is called an *identity permutation* and simply denoted by **1** = (1)(2)⋯(*n*).

The *composition* (or *product*) of two given permutations *α* and *β* of *E* is a permutation, denoted by *αβ*, such that *αβ*(*e*) = *α*(*β*(*e*)) for all *e* ∈ *E.* For example, suppose that *α* = (1,6,4)(2,5,3) and *β* = (4,3) are two given permutations of *E* = {1,2, …,6}. Then *αβ* = (1,6,4,2,5,3). It is not hard to see that if *α* and *β* are disjoint, then *αβ* = *βα.* The *inverse* of *α*, denoted by *α*^–1^, is a permutation such that *αα*^–1^ = *α*^–1^*α* = **1**. The *conjugation* of *β* by *α*, denoted by *α* ⋅ *β*, is the permutation *αβα*^–1^.

As demonstrated in [11,17,18], the permutation groups can serve as a useful tool for studying genome rearrangement, because a genome can be expressed using a permutation, in which each cycle corresponds to a chromosome in the genome, and a fusion or a fission acting on the genome can be simulated by the product of a 2-cycle and the corresponding, as detailed as follows. Let *α* = (*a*_1_, *a*_2_) be a 2-cycle and *β* be an any permutation of *E.* If both *a*_1_ and *a*_2_ belong to the same cycle of *β*, then the effect of *αβ* (or *βα*) is equivalent to a fission acting on *β* and hence *α* is called a *split* operation of *β.* For instance, suppose that *α* = (1, 2) and *β* = (1, 6, 4, 2, 5, 3) . Then *αβ* = (1, 6, 4)(2, 5, 3) and *βα* = (5, 3, 1)(6, 4, 2). On the other hand, if *a*_1_ and *a*_2_ belong to two different cycles of *β*, then the effect of *αβ* (or *βα*) equals to a fusion acting on *β* and *α* is called a *join* operation of *β.* For instance, if *α* = (1,2) and *β* = (1, 6, 4)(2, 5, 3), then *αβ* = (1, 6, 4, 2, 5, 3) and *βα* = (6, 4, 1, 5, 3, 2).

In fact, any permutation *α* of *E* can be written as a composition of 2-cycles in many ways [11]. The *norm* of *α*, denoted by ||*α*||, is the minimum number *k* such that *α* can be expressed by a composition of *k* 2-cycles. The number of disjoint cycles in the cycle decomposition of *α* is denoted by *n_c_*(*α*), which needs to count those non-expressed 1-cycles in *α.* For instance, if *α* = (1, 3, 2)(5,6) and *E* = {1, 2, …,6}, then *n_c_*(*α*) = 3, rather than *n_c_*(*α*) = 2, because *α* = (1, 3, 2)(4)(5, 6). For any permutation *α* of *E*, it can be shown that ||*α*|| = |*E*| – *n_c_*(*α*) [*11*,*17*]. For any two permutations *α* and *β* of *E*, *α divides β*, denoted by *α|β*, if and only if ||*βα*^–1^|| = ||*β*|| – ||*α*||. Actually, whether *α* divides *β* or not can be easily determined using the following lemma from [11].

**Lemma 1**[11]. *Let e*_1_, *e*_2_, *…*, *e_k_* ∈ *E and β be any permutation of E. Then e*_1_, *e*_2_, *…*,*e_k_ appear in the same cycle of β in the order of e*_1_, *e*_2_, *…*, *e_k_ if and only if* (*e*_1_, *e*_2_, …, *e_k_*)*|β.*

It is required to further extend the definition of *E* as *E* = {±1, ±2, …, ±*n*} for properly modeling reversals using the permutation groups, as described in Lemma 3 below. Let Γ = (1, –1)(2, –2) ··· (*n*, –*n*). It is not difficult to verify that Γ^2^ = **1** and Γ^–1^ = Γ. If a cycle contains no *e* and –*e* at the same time, where *e* ∈ *E*, then it is called *admissible* and can be used to denote a DNA strand. Let *π*^+^ denote a strand of a DNA molecule *π*. Then *π*^–^ = Γ · (*π*^+^)^–1^ is the *reverse complement* of *π*^+^, representing another strand of *π*. Note that *π*^+^ and *π*^–^ are disjoint. For the purpose of modeling reversals using the permutation groups, the DNA molecule *π* is represented by the composition of its two strands *π*^+^ and *π*^–^ (i.e., *π* = *π*^+^*π^–^* = *π*^–^*π*^+^), as demonstrated in [11].

**Lemma 2 **[11]. *Let π and σ be two different chromosomes. Suppose that α is a cycle in σπ*^–1^*. Then* (*πΓ*) *· α*^–1^*is also a cycle in σπ*^–1^.

Actually, *α* and (*π*Γ) · *α*^–1^ are *mate cycles* for each other in *σπ*^–1^ according to Lemma 2.

**Lemma 3**[11]. *Let u and v be in the different strands of a chromosome π*, *that is*, (*u*, *v*) ł *π. Then γ* = (*πΓ*(*v*), *πΓ*(*u*)) (*u*, *v*) *affects π as a reversal.*

Note that in Lemma 3, (*u*, *v*) acts on *π* as a join operation and (*π*Γ(*v*),*π*Γ(*u*)) acts on (*u*, *v*)*π* as a split operation, indicating that a reversal acting on *π* can be implemented using the product of two 2-cycles and *π.* Actually, other non-reversal CCLP operations can be implemented by multiplying four 2-cycles (*π*Γ(*x*),*π*Γ(*w*))(*w*, *x*)(*π*Γ(*v*),*π*Γ(*u*))(*u*, *v*) with the given chromosome *π* if the following conditions are satisfied: (1) (*u*, *v*)*|π*, (2) (*w*, *x*) ł (*u*, *v*)*π* (3) *w* ≠ Γ(*x*) or Γ(*w*) ≠ *x* and (4) (*w*, Γ(*x*)) ł (*u*, *v*)*π* or (Γ(*w*), *x*) ł (*u*, *v*)*π.* The first condition is to make sure that (*u*, *v*) and (*π*Γ(*v*),*π*Γ(*u*)) respectively act on the two strands of *π* as splits, which lead to two temporary circles excised from *π.* Note that these two temporary circles are complement to each other. The second condition is to make sure that (*w*, *x*) and (*π*Γ(*x*), *π*Γ(*w*)) respectively act on the two temporary circles and the cycles of the remaining *π* as joins, which paste back the two temporary circles into the remaining *π.* It is worth mentioning that the joins also fulfill the process of linearization with possible inversion. The inversion is performed when the temporary circles are reinserted into the chromosome strands different from the ones they come from. The third and fourth conditions are to make sure that the resulting *π* are admissible (i.e., no *e* and –*e* from *E* are in the same chromosome strand). Therefore, we have the following lemma.

**Lemma 4. ***Let π be a chromosome and β* = (*πΓ*(*x*), *πΓ*(*w*))(*w*, *x*)(*πΓ*(*v*),*πΓ*(*u*))(*u*, *v*)*. Suppose that the following four conditions are satisfied:* (1) (*u*, *v*)*|π*, (2) (*w*, *x*) ł (*u*, *v*)*π* (3) *w* ≠ *Γ*(*x*) *or Γ*(*w*) ≠ *x and* (4) (*w*, *Γ*(*x*)) ł (*u*, *v*)*π or* (*Γ*(*w*), *x*) ł (*u*, *v*)*π. Then β affects π as a non-reversal CCLP operation.*

## Algorithmic result

In this section, we design an efficient algorithm on the basis of the permutation groups that sorts a given chromosome *π* into *I* = (1, 2, *…*, *n*)(*–n*, *…*, –2, –1) using the CCLP operations when the weight ratio between reversals and non-reversal CCLP operations is 1:2. The basic idea behind this algorithm is as follows. As mentioned before, any permutation can be written as a product of 2-cycles and the effect of a reversal (respectively, non-reversal CCLP operation) acting on *π* can be simulated by multiplying two (respectively, four) 2-cycles with *π.* Moreover, the product of *Iπ*^–1^ and *π* equals to *I*. All these facts indicate that one can derive a product of 2-cycles from *Iπ*^–1^ such that these 2-cycles perform as a sequence of CCLP operations to optimally transform *π* into *I*. Below, for simplicity of describing our algorithm, *x* and *y* are said to be *adjacent* in a permutation *α* if *α*(*x*) = *y* or *α*(*y*) = *x*.

**Lemma 5. ***Let π* = *π*^+^*π*^–^*be a chromosome. Suppose that* (*x*, *y*)*|Iπ*^–1^*and* (*x*, *y*)|*π*, *that is*, *there are two elements x and y in a cycle of Iπ*^–1^*such that* (*x*, *y*) *acts on π as a split. Let β* = (*π*Γ(*y*), *π*Γ(*x*))(*x*, *y*)*. Then there are two adjacent elements x*′ *and y*′ *in a cycle of I*(*βπ*)^–1^*such that* (*x*′ ,*y*′) *and* (*βπ*Γ(*y*′),*βπ*Γ(*x*′)) *act on βπ as joins. Moreover*, *the cycles in β*′*βπ are admissible*, *where β*′ = (*βπ*Γ(*y*′), *βπ*Γ(*x*′))(*x*′ ,*y*′).

*Proof.* For convenience, let *π* = *π*^+^*π^–^* = (*a*_1_, *a*_2_, *… a_n_*)(–*a_n_*, –*a_n_*_–1_, …, –*a*_1_). The assumption (*x*, *y*)|*π* indicates that *x* and *y* are in the same cycle of *π*, say in *π*^+^, and hence *π*Γ(*x*) and *π*Γ(*y*) are in *π*^–^. Hence, both (*x*, *y*) and (*π*Γ(*y*),*π*Γ(*x*)) act on *π* as splits and *β* = (*π*Γ(*y*), *π*Γ(*x*))(*x*, *y*) divides *π* into four cycles. Let . For simplicity of our further discussion, we assume that *a_i_* <*a_i_*_+1_ <*n* for 1 ≤ *i* ≤ *k –* 2. This indicates that *a_k_*_–1_ is the maximum in  and hence *a_k_*_–1_ + 1 is not in . Moreover, *I*(*βπ*)^–1^(*a*_1_) = *I*(*a_k_*_–1_) = *a_k_*_–1_ + 1, meaning that *a*_1_ and *a_k_*_–1_ + 1 are adjacent in *I*(*βπ*)^–1^. In other words, there are two adjacent elements *a*_1_ and *a_k–_*_1_ + 1 in *I*(*βπ*)^–1^ such that (*a*_1_,*a_k_*_–1_ + 1), as well as (*βπ*Γ(*a_k_*_–1_ + l), *βπ*Γ((*a*_1_)), acts on *βπ* as a join. If the two cycles in (*βπ*Γ(*a_k_*_–1_ + 1),*βπ*Γ(*a*_1_))(*a*_1_, *a_k_*_–1_ + 1)*βπ* are admissible (i.e., they represent a chromosome), then we have completed the proof of this lemma based on Lemma 4. Now, suppose that the two cycles in (*βπ*Γ(*a_k_*_–1_ + 1),*βπ*Γ(*a*_1_))(*a*_1_, *a_k_*_–1_ + l)*βπ* are not admissible (i.e., for some 1 ≤ *i* ≤ *n*, both *i* and –*i* are in the same cycle). We then show below that we can still find two other adjacent elements *x*′ and *y*′ in a cycle of *I*(*βπ*)^–1^ such that (*x*′ ,*y*′) and (*βπ*Γ(*y*′),*βπ*Γ(*x*′)) can join *βπ* into two admissible cycles. First of all, *a_k_*_–1_ + 1 must be in  (otherwise, (*βπ*Γ(*a_k_*_–1_ + 1),*βπ*Γ(*a*_1_))(*a*_1_,*a_k_*_–1_ + 1)*βπ* is an admissible chromosome), leading to that the cycle created by joining  using (*a*_1_, *a_k_*_–1_ + 1) is not admissible. Further suppose that *a_j_* is the minimum in . Then Γ(*a_j_*) = –*a_j_*, which is the maximum in . Therefore, we have –*a_j_* ≥ *a_k_*_–1_ + 1 (since *a_k_*_–1_ + 1 is also in ). In addition, –*a_j_*_–1_ and *I*(–*a_j_*) are adjacent in *I*(*βπ*)^–1^ because *I*(*βπ*)^–1^(*–a_j_*_–1_) = *I*(–*a_j_*). In the following, we consider five possibilities.

**Case 1.*** a_j_* ≠ –*n* and *a_j_* ≠ 1. Then *I*(–*a_j_*) = –*a_j_* + 1, which is not in  since –*a_j_* is the maximum in . If –*a_j_* + 1 is in , then *a_k_*_–1_ cannot be the maximum in , since –*a_j_* ≥ *a_k_*_–1_ + 1 and hence –*a_j_* + 1 >*a_k_*_–1_ which contradicts to our assumption that *a_k_*_–1_ is the maximum in . In other words, *I*(–*a_j_*) belongs to either  or  and hence (–*a_j_*_–1_, *I*(–*a_j_*)) acts on *βπ* as a join and the cycles in (*βπ*Γ*I*(–*a_j_*),*βπ*Γ(–*a_j_*_–1_))(*–a_j_*_–1_,*I*(–*a_j_*))*βπ* are admissible.

**Case 2.*** a_j_* = –*n* and both 1 and –1 are not in . Then *I*(–*a_j_*) = 1 (instead of *I*(–*a_j_*) = –*a_j_* + 1 = *n* + 1). Because  and  are complement to each other from chromosomal point of view, both of them contains no 1 and –1, as a result, *I*(–*a_j_*) belongs to either  or . Therefore, (–*a_j_*_–1_,*I*(–*a_j_*)) acts on *βπ* as a join and (*βπ*Γ*I*(–*a_j_*),*βπ*Γ(–*a_j–_*_1_))(–*a_j_*_–1_, *I*(–*a_j_*))*βπ* contains only admissible cycles.

**Case 3. ***a_j_* = 1 and both *n* and –*n* are not in . Then *I*(–*a_j_*) = –*n* (instead of *I*(–*a_j_*) = –*a_j_* + 1 = 0 ). Clearly, *I*(–*a_j_*) belongs to either  or . Therefore, (–*a_j_*_–1_,*I*(–*a_j_*)) acts on *βπ* as a join and (*βπ*Γ*I*(–*a_j_*), *βπ*Γ(–*a_j_*_–1_))(–*a_j_*_–1_, *I*(–*a_j_*))*βπ* have two admissible cycles.

**Case 4.*** a_j_* = –*n* and 1 or –1 is in . Because  and  are complement strands, 1 is in  if and only if –1 is in . Hence, both  and  contains no –*n*, 1 and –1. Then we can exchange the roles of  and  with  and , respectively, and follow the similar discussion as given in Case 1 to show that we can still find two adjacent elements *x*′ and *y*′ in a cycle of *I*(*βπ*)^–1^ such that (*x*′ ,*y*′) and (*βπ*Γ(*y*′), *βπ*Γ(*x*′)) can join the four cycles of *βπ* into two admissible cycles.

**Case 5. ***a_j_* = 1 and *n* or –*n* is in . Actually, we need not consider this case, because we have initially assumed that all the elements in  are less than *n* and among them, *a_j_* is the smallest.

According to the above discussion, we have completed the proof of this lemma.

**Theorem 1.*** Let* Φ *denote a minimum weighted sequence of CCLP operations required to transform π into I. Then the weight of* Φ *is great than or equal to*.

*Proof.* Let Φ contain *a* reversals and *b* non-reversal CCLP operations. It is not hard to see that *a* + 2*b* is the weight of Φ. Recall that the effect of a reversal can be simulated using two 2-cycles and a non-reversal CCLP operation using four 2-cycles. It indicates that Φ can be written by a composition of 2*a* + 4*b* 2-cycles such that Φ*π* = *I*, which equals to that *Iπ*^–1^ can be expressed as a composition of 2*a* + 4*b* 2-cycles. In other words, ||*Iπ*^–1^|| ≤ 2*a* + 4*b*. As mentioned before, we also have ||*Iπ*^–1^|| = |*E*| – *n_c_*(*Iπ*^–1^), which bases on the lemma proposed in [11,17]. Therefore, |*E*| – *n_c_*(*Iπ*^–1^) ≤ 2*a* + 4*b* and, as a result, the weight of Φ is great than or equal to .

Assume that there are at least two adjacent elements *x* and *y* in a cycle of *Iπ*^–1^ such that (*x*, *y*)|*π*. Then, according to Lemma 5, we can always find a non-reversal CCLP operation *β*′*β* from *Iπ*^–1^ to rearrange *π* into *β*′*βπ*, where *β* = (*π*Γ(*y*), *π*Γ(*x*))(*x*, *y*) and *β*′ = (*βπ*Γ(*y*′),*βπ*Γ(*x*′))(*x*′, *y*′). Assume that there are no any two adjacent elements *x* and *y* in a cycle of *Iπ*^–1^ such that (*x*, *y*)|*π*, which implies that (*x*, *y*) ł *π.* Then based on Lemma 3, (*π*Γ(*y*), *π*Γ(*x*))(*x*,*y*) can serve as a reversal to transform *π* into (*π*Γ(*y*), *π*Γ(*x*))(*x*, *y*)*π*. Using these properties, we design Algorithm 1 to sort *π* into *I by* CCLP operations. It is not hard to see that a non-reversal CCLP operation derived in Algorithm 1 decreases the norm of *Iπ*^–1^ by 4 and a reversal by 2. Since non-reversal CCLP operations are weighted 2 and reversals are weighted 1, Algorithm 1 decreases the norm of *Iπ*^–1^ by 1 at the weight of  and hence its total weight equals to , which is optimal according to Theorem 1.

**Theorem 2. ***Given a chromosome π*, *the weighted sorting problem by CCLP operations can be solved in O*(*δn*) *time when with weight ratio between reversals and non-reversal CCLP operations is* 1:2, *where δ is the number of CCLP operations needed to transform π into I. Moreover*, *the weight of the optimal solution is**that can be calculated in O*(*n*) *time in advance.*

*Proof.* As discussed before, Algorithm 1 transforms *π* into *I* by a minimum weighted sequence of *δ* CCLP operations, whose total weight is  that can be calculated in *O*(*n*) time. Below, the time-complexity of Algorithm 1 is analyzed. Basically, the computation in steps 1 and 2 can be done in *O*(*n*) time. As for step 3, there are *δ* iterations to perform. For each such iteration, it takes *O*(*n*) time to find (*x*, *y*) and (*x*′, *y*′) by determining every pair of adjacent elements in all the cycles of *Iπ*^–1^ and *Iπ*^–1^*β*, respectively, and a constant time to perform other operations in step 3.1, and also takes *O*(*n*) time to perform step 3.2. Therefore, the cost of step 3 is *O*(*δn*). Step 4 is executed in constant time. Totally, the time-complexity of Algorithm 1 is *O*(*δn*).

It is worth mentioning here that our algorithm is applicable to both circular and linear chromosomes. Actually, using similar discussion as in [17], one can prove that given a gene *x* on a circular chromosome, a CCLP operation acting on *x* has an equivalent one without acting on *x*. Based on this property, one can further prove that the problem of sorting by CCLP operations is equivalent for circular and linear chromosomes.

## Conclusion

In this article, we have introduced and studied the sorting problem by CCLP operations, where CCLP is a cut-circularize-linearize-and-paste operation that can model several known and unknown rearrangements. In addition, we have proposed an *O*(*δn*) time algorithm for solving the weighted sorting problem by CCLP operations when the weight ratio between reversals and non-reversal CCLP operations is 1:2, where *n* is the number of genes and *δ* is the number of needed CLLP operations. As described in this article, this algorithm is very simple so that it can be easily implemented using 1-dimensional arrays and useful in the studies of phylogenetic tree reconstruction and human immune response to tumors. It would be an interesting future work to design efficient algorithms for solving the problem of sorting by CCLP operations when all the CCLP operations are weighted equally.

## Competing interests

The authors declare that they have no competing interests.

## Authors’ contributions

CLL conceived of this study, designed and analyzed its algorithm and drafted the manuscript. KHH and KTC participated in the design and analysis of the algorithm and the draft of the manuscript. All authors read and approved the final manuscript.

## References

[B1] Sankoff D Leduc G Antoine N Paquin B Lang BF Cedergren R Gene order comparisons for phylogenetic inference: evolution of the mitochondrial genome Proceedings of the National Academy of Sciences 1992 89 6575 6579 10.1073/pnas.89.14.6575 PMC49544 1631158

[B2] Hannenhalli S Pevzner PA Transforming men into mice (polynomial algorithm for genomic distance problem) Proceedings of the 36th IEEE Symposium on Foundations of Computer Science (FOCS 1995) 1995 IEEE Computer Society 581 592

[B3] Hannenhalli S Pevzner PA Transforming cabbage into turnip: polynomial algorithm for sorting signed permutations by reversals Journal of the ACM 1999 46 1 27 10.1145/300515.300516

[B4] Pevzner P Tesler G Genome rearrangements in mammalian evolution: lessons from human and mouse genomes Genome Research 2003 13 37 45 10.1101/gr.757503 12529304 PMC430962

[B5] Belda E Moya A Silva FJ Genome rearrangement distances and gene order phylogeny in γ-Proteobacteria Molecular Biology Evolutionary 2005 22 1456 1467 10.1093/molbev/msi134 15772379

[B6] Huang YL Huang CC Tang CY Lu CL SoRT^2^: a tool for sorting genomes and reconstructing phylogenetic trees by reversals, generalized transpositions and translocations Nucleic Acids Research 2010 38 W221 W227 10.1093/nar/gkq520 20538651 PMC2896082

[B7] Kaplan H Shamir R Tarjan RE Faster and simpler algorithm for sorting signed permutations by reversals SIAM Journal on Computing 1999 29 880 892

[B8] Bader DA Moret BM Yan M A linear-time algorithm for computing inversion distance between signed permutations with an experimental study Journal of Computational Biology 2001 8 483 491 10.1089/106652701753216503 11694179

[B9] Tannier E Bergeron A Sagot MF Advances on sorting by reversals Discrete Applied Mathematics 2007 155 881 888 10.1016/j.dam.2005.02.033

[B10] Bader M Ohlebusch E Sorting by weighted reversals, transpositions, and inverted transpositions Journal of Computational Biology 2007 14 615 636 10.1089/cmb.2007.R006 17683264

[B11] Huang YL Lu CL Sorting by reversals, generalized block-interchanges, and translocations using permutation groups Journal of Computational Biology 2010 17 685 705 10.1089/cmb.2009.0025 20500022

[B12] Bafna V Pevzner PA Sorting by transpositions SIAM Journal on Discrete Mathematics 1998 11 221 240

[B13] Meidanis J Dias Z Navarro G Genome rearrangements distance by fusion, fission, and transposition is easy Proceedings of the 8th International Symposium on String Processing and Information Retrieval (SPIRE 2001) 2001 IEEE Computer Society 250 253

[B14] Elias I Hartman T Casadio R and Myers G A 1.375-approximation algorithm for sorting by transpositions Proceedings of the 5th Work shop on Algorithms in Bioinformatics (WABI 2005), Volume 3692 of Lecture Notes in Computer Science 2005 Springer-Verlag 204 215

[B15] Feng JX Zhu DM Faster algorithms for sorting by transpositions and sorting by block interchanges ACM Transactions on Algorithms 2007 3 3 10.1145/1273340.1273341

[B16] Christie DA Sorting by block-interchanges Information Processing Letters 1996 60 165 169 10.1016/S0020-0190(96)00155-X

[B17] Lin YC Lu CL Chang HY Tang CY An efficient algorithm for sorting by block-interchanges and its application to the evolution of vibrio species Journal of Computational Biology 2005 12 102 112 10.1089/cmb.2005.12.102 15725736

[B18] Lu CL Huang YL Wang TC Chiu HT Analysis of circular genome rearrangement by fusions, fissions and block-interchanges BMC Bioinformatics 2006 7 295 10.1186/1471-2105-7-295 16768797 PMC1569878

[B19] Huang YL Huang CC Tang CY Lu CL An improved algorithm for sorting by block-interchanges based on permutation groups Information Processing Letters 2010 110 345 350 10.1016/j.ipl.2010.03.003

[B20] Hannenhalli S Polynomial algorithm for computing translocation distance between genomes Discrete Applied Mathematics 1996 71 137 151 10.1016/S0166-218X(96)00061-3

[B21] Bergeron A Mixtacki J Stoye J On sorting by translocations Journal of Computational Biology 2006 13 567 578 10.1089/cmb.2006.13.567 16597257

[B22] Ozery-Flato M Shamir R Lewenstein M and Valiente G An algorithm for sorting by reciprocal translocations Proceedings of the 17th Annual Symposium on Combinatorial Pattern Matching (CPM 2006), Volume 4009 of Lecture Notes in Computer Science 2006 Springer 258 269

[B23] Yancopoulos S Attie O Friedberg R Efficient sorting of genomic permutations by translocation, inversion and block-interchanges Bioinformatics 2005 21 3340 3346 10.1093/bioinformatics/bti535 15951307

[B24] Adam Z Sankoff D The ABCs of MGR with DCJ Evol Bioinform Online 2008 4 69 74 19204809 PMC2614205

